# Clinicopathological study on camel mastitis at Matrouh Governorate

**DOI:** 10.5455/javar.2023.j680

**Published:** 2023-06-30

**Authors:** Asmaa Abdallah Darwish

**Affiliations:** Animal and Poultry Health Department, Animal and Poultry Health Division, Desert Research Center, Cairo, Egypt

**Keywords:** Bacterial causes, biomarkers, camel mastitis, clinicopathological alterations, immunological alterations, iron profile

## Abstract

**Objective:**

Camel mastitis is indeed a serious problem that can have significant impacts on animal health and production as well as pose a potential public health hazard. This work aimed to identify the bacterial species responsible for camel mastitis and evaluate the associated immunological and clinicopathological alterations in infected camels.

**Materials and Methods:**

Raw milk and blood samples were collected from 40 apparently healthy she-camels, and 40 she-camels suffered from clinical mastitis (CMG). Milk samples were subjected to bacteriological examination. Serum immunological, biochemical, and hematological parameters were estimated and statistically analyzed.

**Results:**

Similar bacterial species were obtained from the two groups with different isolation rates.*Staphylococcus epidermidis* and *Escherichia coli*were the dominant species in the apparently healthy group, while*Pseudomonas aeruginosa*and*Bacillus cereus* were the dominant species in CMG. A significant (*p *< 0.05) elevation of the pro-inflammatory cytokines, acute phase proteins (APPs), free radicals, total protein, Glob, kidney and liver function tests, and triglyceride concentrations were detected in CMG, and a significant (*p *< 0.05) decrease in the anti-inflammatory cytokine, antioxidants, Alb, glucose, and T/LDL/HDL-cholesterol concentrations was observed in CMG. Microcytic hypochromic anemia with hypoferremia, hypotransferrinemia, hyperferritinemia, and neutrophilic leukocytosis was depicted in CMG. The estimated pro-inflammatory cytokines, APPs, and total antioxidant capacity (TAC) yielded high sensitivity and specificity, but the highest likelihood ratio was for TAC, fibrinogen (Fb), and ferritin, and the highest percentages of increase were for IL-1α and IL-1β.

**Conclusion:**

The study emphasizes the importance of hygienic preventive measures to control camel mastitis and the importance of supportive treatment to reverse the hemato-biochemicaland iron profile changes that result from the immune response in mastitic she-camels. TAC, Fb, ferritin, IL-1α, and IL-1β are good biomarkers for camel mastitis.

## Introduction

The camel (*Camelus dromedarius*) is an exceptional species that has developed remarkable adaptations to endure water scarcity and sustain itself by consuming thorny vegetation. In arid and semi-arid regions, camels serve as valuable sources of both meat and milk. Additionally, camels have been historically utilized as a means of transportation [1–3]. The milk produced by camels possesses nutritional qualities akin to those of other dairy animals’ milk, encompassing all essential nutrients. Notably, camel milk exhibits heightened mineral content, specifically calcium, sodium, magnesium, iron, and copper, while containing lower levels of sugar and cholesterol than cow milk. Moreover, camel milk contains approximately two to three times more vitamin C than cow milk. This elevated vitamin C content holds significant significance in regions characterized by arid and semi-arid climates, where the availability of fruits and vegetables is scarce [4–6]. Furthermore, vitamin C contributes to the preservation of camel milk by increasing its acidity, thus extending its shelf life. Remarkably, camels continue to produce milk even under stressful circumstances like drought, while other milk-producing animals cease lactation. Camels exhibit an extended duration of lactation, yielding around 5–6 l of milk per day, even during periods of drought [1–3].

Mastitis, an inflammation of the mammary gland, is widely recognized as a significant economic concern in the dairy industry worldwide. It affects various domesticated animal species and is prevalent across diverse geographical locations [1,6]. This disease leads to substantial financial losses, including reduced milk production, expenses associated with treatment and veterinary services, and milk disposal. Furthermore, mastitis poses risks to both human health and suckling calves due to the potential presence of highly pathogenic organisms such as *Mycobacterium* and *Brucella* in the milk [4–6]. Multiple factors can contribute to the development of mastitis, including bacterial infections, udder injuries, or obstructions in the milk ducts. Typical symptoms of mastitis in camels encompass udder swelling and increased temperature, udder pain or discomfort, decreased milk production, and alterations in the milk’s appearance or consistency. In severe instances, the affected area may exhibit redness and a heightened temperature upon touch. Additionally, affected camels may display signs of fever and lethargy [1–3].

Despite extensive research on camel mastitis, all previous works focused on its etiology, epidemiology, and treatment. There is a lack of comprehensive information regarding the immunological and clinicopathological changes associated with this disease. Understanding these changes is crucial for developing effective management strategies that can reduce the morbidity and mortality rates of the disease. Therefore, the aim of this study was to identify the specific bacterial species responsible for camel mastitis and examine the associated immunological and clinicopathological alterations in infected camels. The study also explored the potential utility of pro-inflammatory cytokines, acute phase proteins (APPs), and total antioxidant capacity (TAC) as biomarkers for diagnosing and monitoring camel mastitis.

## Materials and Methods

### Ethical approval 

After the ethical approval No. 1, January 2023, of the animal and poultry health department, animal and poultry production division, DRC, Cairo, Egypt, and owners` agreements, this study was performed on 80 she-camels randomly collected from different cities of Matrouh governorate. Each she-camel was subjected to a general clinical examination and udder examination, and then raw milk and blood samples were obtained from them. The California mastitis test (CMT) was applied to all milk samples according to Schalm et al. [7], and animals were grouped as follows:

*Control group (CG*): 40 apparently healthy she-camel with normal ranges of body temperature, pulse rate, and respiratory rate. Normal udder texture and milk samples were negative for CMT (the mixture remained liquid).

*Clinical mastitic group (CMG):* 40 she-camels suffered from anorexia, hyperthermia, a high pulse, and respiratory rates. The udder was swollen, congested, hot, and painful upon examination. A significant drop in milk production, with abnormal appearance and consistency. Milk samples were positive for CMT (the mixture thickened immediately and tended to form jelly).

### Bacteriological examination

Raw milk samples were aseptically inoculated with a loopful into the nutrient and selenite cystine broths. Incubation was carried out at 37°C for 24 h. Subsequently, a loopful of the nutrient broth culture was streaked onto various types of agar, including nutrient agar, MacConkey’s agar, brain heart infusion agar, mannitol salt agar, and litmus milk media, for the purpose of bacterial isolation. Another loopful from the selenite cystine broth culture was inoculated onto SS agar to isolate *Salmonella* and *Enterococcus* species. After incubation at 37°C for 24 h, morphological and biochemical identification of the isolated bacterial strains was conducted according to the guidelines provided by Quinn et al. [8]. Isolation of *Acholeplasma* from milk samples was achieved using *Mycoplasma* agar and broth supplemented with *Mycoplasma *selective supplement G, following the procedures described by Hazelton et al. [9].

### Clinicopathological examination 

The blood samples were divided into three parts for analysis. The first part was collected using Na_2_EDTA and used for manual estimation of the hemogram [red blood cell (RBCs), hemoglobin concentration (Hb), packed cell volume (PCV), mean corpuscular volume (MCV), mean corpuscular hemoglobin (MCH), and mean corpuscular hemoglobin concentration (MCHC)] and leukogram [total leukocytic count (TLC) and differential leukocytic count] parameters of animals in both groups according to the method described by Feldman et al. [10]. The second part was collected on citrate to prevent the coagulation process and centrifuged to obtain plasma at 3,000 rpm for 20 min to measure fibrinogen (Fb) plasma levels using ELISA kits from IBL International Crop (Canada)^®^. The third part was collected in a plain test tube, allowed to coagulate, and centrifuged at 3,000 rpm for 20 min to obtain serum for detection of various parameters including total protein (TP), Glob, kidney function tests [urea, creatinine (Cr)], hepatic enzymes [alanine aminotransferase (ALT), aspartate aminotransferase (AST), gamma-glutamyl transferase (GGT), alkaline phosphatase (ALP)], minerals (Ca, P, Mg), electrolytes (Na, K, Cl), trace elements (Cu, Zn), glucose, total lipids, phospholipids, triglycerides, T/LDL/HDL-cholesterol, serum concentrations of free radicals [nitric oxide (NO), malondialdehyde (MDA), hydrogen peroxide (H_2_O_2_)], TAC, antioxidants [catalase (CAT), glutathione perox (GPx), glutathione reductase (GR)], Serum iron (SI) and total iron binding capacity (TIBC) spectrophotometrically using kits from Biodiagnostic Company^®^, serum pro-inflammatory cytokines (IL-1α, IL-1β, IL-6, TNF-α) and anti-inflammatory cytokine (IL-10) using ELISA kits from MyBioSource Company^®^, serum amyloid A (SAA) and serum haptoglobin (Hp) using ELISA kits from IBL International Crop (Canada)^®^, Serum caeruloplasmin (Cp) and serum transferrin (Tf) by a turbidimetric method using Elabscience USA^®^ kits, and serum ferritin by CLIA method using Abnova^®^ (Taipei) kits. All manual instructions were carefully followed.

Tf saturation percent (TF sat.%) = SI/TIBC*100

Unsaturated iron binding capacity (UIBC) = TIBC-SI

### Statistical analysis

The mean values of CG and CMG were compared by an independent-sample *t*-test using SPSS^®^ program version 23. A difference was considered significant at *p* < 0.05. The GraphPad Prism version 8 program was used to evaluate the area under the curve (AUC), cut-off points, sensitivity, specificity, and likelihood ratio (LR) for the measured cytokines, APPs, and TAC between CMG and CG. The positive predictive value (PPV), negative predictive value (NPV), and accuracy rate (AR) for them were calculated according to the following equations:

PPV = True positive ÷ Total positive × 100.

NPV = True negative ÷Total negative × 100.

Accuracy rate = (True positive + True negative) ÷ Total population × 100.

Percentages of increase or decrease for estimated markers = (The mean value of the marker concentration in CMG - The mean value of its concentration in CG) ÷ The mean value of its concentration in CG ×100.

## Results

### Bacteriological results

In the CG, a total of 40 bacterial isolates were retrieved from 40 milk samples, with each sample yielding a single isolate. The most frequently identified isolate was *Staphylococcus epidermidis*, comprising 40% (16/40) of the isolates, followed by *Escherichia coli* at 17.5% (7/40). *Pseudomonas aeruginosa* and *Klebsiella pneumonia* were found in 10% (4/40) of the samples, while *Proteus vulgaris *and *Shigella flexneri* were identified in 5% (2/40) of the samples. The remaining isolates, each representing 2.5% (1/40) of the total, were *Enterobacter aerogenes*, *Serratia marcescens*, *Streptococcus agalactiae*, *Bacillus cereus*, and *Enterococcus faecalis *(Table 1)..

Meanwhile, in the mastitic group, a total of 110 bacterial isolates were recovered from 40 milk samples, with 30 cases yielding 3 isolates each and 10 cases yielding 2 isolates each. The most frequently retrieved isolate was *P. aeruginosa* at a rate of 22.73% (25/110), followed by *B. cereus* at 16.36% (18/110) and *S. epidermidis* at 15.45% (17/110). *E. coli* accounted for 12.73% (14/110) of the isolates, while *K. pneumoniae* and *S. flexneri* were present in 7.27% (8/110) of the total isolates. *Staphylococcus aureus* had a recovery rate of 4.55% (5/110), while *E. aerogenes* and *E. faecalis* had isolation rates of 2.73% (3/110). *Proteus vulgaris, S. marcescens, Yersinia enterococci, and Salmonella typhimurium* were all retrieved at a rate of 1.82% (2/110). Finally, *Acholeplasma laidlawii* was identified in a single isolate at a recovery rate of 0.91% (Table 1).

**Table 1.** Prevalence rate of bacterial isolates from apparently healthy and mastitic she-camels.

Bacterial species	CG		CMG	
	No. of bacterial isolates	%	No. of bacterial isolates	%
*Escherichia coli *	7	17.5	14	12.73
*Pseudomonas aeruginosa*	4	10	25	22.73
*Klebsiella pneumonia*	4	10	8	7.27
*Proteus vulgaris*	2	5	2	1.82
*Shigella flexneri*	2	5	8	7.27
*Enterobacter aerogenes*	1	2.5	3	2.73
*Serratia marcescens*	1	2.5	2	1.82
*Yersinia enterococci*	0	0	2	1.82
*Salmonella typhimurium*	0	0	2	1.82
*Staphylococcus epidermidis*	16	40	17	15.45
*Streptococcus agalactiae*	1	2.5	0	0
*Bacillus cereus*	1	2.5	18	16.36
*Enterococcus faecalis*	1	2.5	3	2.73
*Staphylococcus aureus*	0	0	5	4.55
*Acholeplasma laidlawii *	0	0	1	0.91
Total bacterial isolates	40		110	

### Clinicopathological results

A considerable innate immune response was reported in CMG when compared to CG, as represented by the significant (*p* < 0.05) elevation of the pro-inflammatory cytokines (IL-1α, IL-1β, IL-6, TNF-α), APPs (Fb, Cp, SAA, Hp), and free radicals (MDA, NO, H_2_O_2_) concentrations in CMG (in relation to CG) and the significant (*p* < 0.05) decrease of anti-inflammatory cytokine (IL-10), TAC, and antioxidants (CAT, GR, GPx) concentrations in CMG (in relation to CG). A significant (*p* < 0.05) increase was observed in serum levels of TP, Glob, kidney and liver function tests, and triglycerides in CMG in relation to CG, while a significant (*p* < 0.05) decline was detected in serum concentrations of Alb, A/G, Ca, P, Mg, Na, K, Cl, Cu, Zn, glucose, and T/LDL/HDL-cholesterol (Table 2).

The hemogram of the mastitic she-camels showed a significant (*p* < 0.05) decrease in the RBC parameters (RBCs, Hb, and PCV) and indices (MCV, MCH, and MCHC) when compared to the CG hemogram. The iron profile of CMG (in relation to CG) cleared a significant (*p* < 0.05) hypoferremia, hypotransferrinemia, and hyperferritinemia, a significant (*p* < 0.05) increase in TIBC and UIBC, and a significant reduction in Tf sat.%. While significant (*p* < 0.05) leukocytosis, neutrophilia, eosinophilia, monocytosis, and lymphopenia were observed in CMG when compared to CG (Table 3),

**Table 2.** The immunological and biochemical parameters of the mastitic group (CMG) compared to the control (CG). Value = mean ± SD.

Parameter	CG	CMG
IL-1α (pg/ml)	19.76 ± 1.60	89.56 ± 2.76*
IL-1β (pg/ml)	26.86 ± 3.13	112.89 ± 3.86*
IL-6 (pg/ml)	24.65 ± 1.77	91.69 ± 1.95*
TNF-α (Pg/ml)	27.30 ± 2.66	101.17 ± 1.99*
IL-10 (pg/ml)	91.73 ± 2.51	19.30 ± 1.70*
Fb (mg/dl)	69.71 ± 752	106.21 ± 11.70*
Cp (mg/dl)	6.82 ± 0.52	9.80 ± 0.69*
Hp (gm/dl)	0.21 ± 0.02	0.48 ± 0.10*
SAA (mg/l)	2.68 ± 0.19	4.07 ± 0.43*
MDA (nmol/ml)	2.70 ± 0.56	8.15 ± 0.93*
NO (μmol/l)	17.89 ± 4.62	47.65 ± 10.53*
H_2_O_2 _(μm/l)	281.08 ± 23.20	545.81 ± 32.86*
TAC (Mm/l)	1.34 ± 0.15	0.46 ± 0.05*
CAT (U/l)	370.36 ± 20.33	180.81 ± 37.66*
GPx (mU/l)	38.70 ± 6.02	21.66 ± 085*
GR (ng/ml)	7.65 ± 0.98	5.01 ± 0.29*
AST (U/l)	25.28 ± 2.59	34.50 ± 3.85*
ALT (U/l)	19.96 ± 0.84	28.66 ± 0.28*
GGT (U/l)	14.24 ± 0.98	24.00 ± 0.85*
ALP (U/l)	22.97 ± 1.43	40.94 ± 2.76*
Blood urea (mg/dl)	23.40 ± 1.90	41.19 ± 4.16*
Cr (mg/dl)	0.64 ± 0.10	1.14 ± 0.21*
Glucose (mg/dl)	127.01 ± 14.53	87.01 ± 5.25*
Total lipids (mg/dl)	424.29 ± 11.53	422.11 ± 10.77
Triglycerides (mg/dl)	80.34 ± 6.11	118.01 ± 8.52*
Phospholipids (mg/dl)	160.15 ± 6.08	161.56 ± 5.88
T-cholesterol (mg/dl)	183.80 ± 6.61	142.82 ± 5.01*
HDL-cholesterol(mg/dl)	43.95 ± 2.16	27.24 ± 4.36*
LDL-cholesterol (mg/dl)	89.85 ± 5.97	65.75 ± 3.24*
Ca (mg/dl)	10.99 ± 0.67	8.64 ± 0.28*
P (mg/dl)	3.18 ± 0.22	2.24 ± 0.14*
Cl (mmol/l)	108.98 ± 1.15	98.15 ± 0.37*
Na (mmol/l)	129.85 ± 2.33	97.24 ± 3.18*
K (mmol/l)	4.40 ± 0.28	3.05 ± 0.24*
Mg (mg/dl)	3.48 ± 0.16	2.01 ± 0.51
Cu (μg/dl)	140.76 ± 5.42	94.80 ± 2.36*
Zn (μg/dl)	131.86 ± 8.49	99.27 ± 2.14*
TP (gm/dl)	5.90 ± 0.42	7.66 ± 0.30*
Albumin (gm/dl)	3.87 ± 0.34	2.41 ± 0.30
Globulin (gm/dl)	2.04 ± 0.48	5.24 ± 0.37*
A\G	1.97 ± 0.84	0.47 ± 0.08

Significant differences in the values between the CMG and the C G were indicated by (*) at *p *< 0.05.

**Table 3.** The hematological and iron profile parameters of the mastitic group (CMG) compared to the CG. Value = mean ± SD.

Parameter	CG	CMG
RBCs (×10^6^/μl)	10.68 ± 0.43	8.46 ± 0.50*
Hb (gm/dl)	12.46 ± 0.51	8.25 ± 0.74*
PCV (%)	36.52 ± 1.25	26.69 ± 1.50*
MCV (fl)	34.20 ± 0.67	31.56 ± 0.87*
MCH (pg)	11.67 ± 0.44	9.74 ± 0.59*
MCHC (%)	34.12 ± 1.25	30.88 ± 2.02*
SI (μg/dl)	142.48 ± 3.05	86.89 ± 2.08*
TIBC (μg/dl)	369.26 ± 9.43	383.90 ± 2.96*
UIBC (μg/dl)	226.78 ± 10.20	297.01 ± 4.01*
Tf (mg/dl)	121.38 ± 2.21	91.76 ± 3.68*
Tf Sat. %	38.61 ± 1.36	22.64 ± 0.60*
Ferritin (ng/ml)	34.00 ± 5.36	58.07 ± 6.27*
TLC (×10^3^/μl)	8.50 ± 0.32	12.23 ± 0.52*
Neutrophils (×10^3^/μl)	5.12 ± 0.24	8.65 ± 0.39*
Lymphocytes(×10^3^/μl)	2.57 ± 0.14	2.29 ± 0.22*
Monocytes (×10^3^/μl)	0.40 ± 0.06	0.62 ± 0.04*
Eosinophils (×10^3^/μl)	0.35 ± 0.05	0.61 ± 0.03*
Basophils (×10^3^/μl)	0.002 ± 0.004	0.002 ± 0.004

Significant differences in the values between the CMG and the CG were indicated by (*) at *p *< 0.05.

In regard to the diagnostic and prognostic value of the estimated pro-inflammatory cytokines, APPs, and TAC, Table 4 cleared that all of them yielded high sensitivity, specificity, PPV, NPV, and AR at AUC = 1, but their LRs clarified that: TAC, Fb, and ferritin scored high LRs as 21, 21, 10.5, respectively, followed by IL-1β, Cp, Hp, SAA, TNF-α, Tf with moderate LRs as 7, 5.25, 5.25, 5.25, 5.25, 5.25, respectively. In contrast, IL-1α and IL-6 scored low LRs at 4.20, 4,20, respectively. On the other hand, the highest percentages of increase were for IL-1α (353.24%) and IL-1β (320.29%).

## Discussion 

Mastitis is an inflammatory condition that occurs within the mammary gland and leads to various alterations in the milk’s physical, chemical, and bacterial characteristics. It also causes pathological changes in the udder glandular tissue. This condition has detrimental effects on both human health and animal production [1,6].

**Table 4.** Cut-off points, sensitivity%, specificity%, LR, PPV%, NPV%, AR, and percentages of increase or decrease [% of (+, −)] of the estimated TAC, pro-inflammatory cytokines (IL-1α, IL-1β, IL-6, TNF-α) and APPs (Fb, Cp, Hp, SAA, Tf, ferritin) in the mastitic group compared to the CG.

Parameter	Cut-off	Sensitivity %	Specificity %	LR	PPV%	NPV%	AR	% of (+,−)
TAC (mm/l)	1.15	100	95.24	21	96.67	100	98	−65.67
IL-1α (pg/ml)	21.22	100	76.19	4.20	85.29	100	90	353.24
IL-1β (pg/ml)	31	100	85.71	7	90.62	100	94	320.29
IL-6 (pg/ml)	26.22	100	76.19	4.20	85.29	100	90	271.97
TNF-α (pg/ml)	29.50	100	80.95	5.25	87.88	100	92	270.59
Fb (mg/dl)	79.50	100	95.24	21	96.67	100	98	43.75
Cp (mg/dl)	7.35	100	80.95	5.25	87.88	100	92	43.69
Hp (gm/dl)	0.27	100	80.95	5.25	87.88	100	92	128.57
SAA (mg/l)	2.83	100	80.95	5.25	87.88	100	92	51.87
Tf (mg/dl)	119	100	80.95	5.25	87.88	100	92	−24.40
Ferritin (ng/ml)	41	100	90.48	10.50	93.55	100	96	70.79

LR = 0.5–5: low; LR = 5–10: moderate; LR > 10: high.

In the current data, 100% of the milk samples collected from healthy or mastitic she-camels were positive for bacterial isolation. All the apparently healthy she-camel milk samples had only a single bacterial isolate, while all the mastitic group samples had a mixed infection (75% had 3 isolates, and 25% had 2 isolates). This result agreed with previous authors, who mentioned that bacterial infections are the major factor in most mastitis cases in domestic animals, including camels. Whereas the udder acts as an inhabitant for several bacterial species, and under certain conditions (stress, exhaustion, poor nutrition, etc.), these pathogens can spread during milking, causing the contagious form of the disease. In addition, the bacterial pathogens that live in the animal environment (soil, bedding, water, manure, and calving pads) can infect the udder through abrasions and cracked skin, leading to environmental mastitis [1,6]. This highlights the opportunistic nature of these bacteria and explains why the bacterial species recovered from the two groups were nearly the same.

In order, the bacterial species isolated from the apparently healthy camel milk samples were *S. epidermidis* (40%), *E. coli* (17.5%), *P. aeruginosa *(10%), *K. pneumonia* (10%), *P. vulgaris *(5%),* S. flexneri* (5%), *E. aerogenes* (2.5%), *S. marcescens *(2.5%)*, S. agalactiae* (2.5%), *B. cereus *(2.5%)*, *and *E. faecalis *(2.5%). The bacterial species recovered from the CMG were *P. aeruginosa* (22.73%),* B. cereus* (16.36%), then *S. epidermidis* (15.45%), *E. coli* (12.73%), *K. pneumonia *(7.27%),* S. flexneri* (7.27%), *S. aureus* (4.55%), *E. aerogenes *(2.73%), *E. faecalis* (2.73%), *P. vulgaris *(1.82%), *S. marcescens* (1.82%), *Y. enterococci *(1.82%), *S. typhimurium *(1.82%), and *A. laidlawii *(0.91%). Similar bacterial species were previously recovered (with varying isolation rates) from apparently-healthy, clinical, and subclinical mastitic she-camels by many researchers in Egypt, the UAE, Iraq, Rwanda, Ethiopia, and Kenya [1–6]. The variation in the isolation percentages between the current work and previous research may be due to the differences in the study area, isolation methods, age, and raring systems.

The growth and multiplication of these bacterial species and subsequent metabolic products in the mastitic she-camels udder in this work successfully stimulated a systemic innate immune response. This was indicated by the distinguished increase in the concentrations of the pro-inflammatory cytokines (IL-1α, IL-1β, IL-6, TNF-α) recorded in CMG. Pro-inflammatory cytokines are crucial in the immune response’s initiation, maintenance, and regulation. They are non-specifically secreted by various immune cells in response to different types of infections. They trigger different immune cells and molecules to perform their duties and coordinate between them. Pro-inflammatory cytokines upregulation has been noticed in clinical and subclinical mastitis in dairy cows and ewes [11–15] and different types of bacterial infections and inflammatory conditions in dromedaries [16,17]. Conversely, anti-inflammatory cytokines (Il-10) are the terminator of the inflammatory immune response. They work antagonistically to the pro-inflammatory cytokines to prevent the exaggeration of the immune response. Il-10 decrease in the current research and previous studies was greatly involved in expanding the inflammatory response and the appearance of the disease symptoms [12,18].

The pro-inflammatory cytokines promote APP secretion from the liver. They are non-specific immune proteins that rapidly constrict the pathogen’s spread and multiplication in the host body until specific immunity is formed. Their persistence in the infected animal’s body suggested them as reliable biomarkers for different diseases. This study demonstrated a marked increase in APPs (Fb, Cp, SAA, and Hp) concentrations in CMG. Similar observations were reported before for mastitis in camels and other animal species [15,19–21]. Additionally, the pro-inflammatory cytokines prompt free radicals liberation from different immune cells. Free radicals are another part of the host’s innate immunity, they kill the invading pathogen through the oxidation of its vital components such as DNA, carbohydrates, lipids, and proteins. Free radicals are supposed to be controlled by antioxidants, but in prolonged chronic conditions, free radicals massively accumulate and antioxidants are consumed. This imbalance between free radicals and antioxidants is known as oxidative stress. Paradoxically, oxidative stress is greatly implicated in different diseases’ pathogenesis, including mastitis, as the uncontrolled free radicals react with the host body cells and oxidize and destroy them instead of the pathogen cells. Oxidative stress was represented here by the pronounced increase in free radical concentrations (MDA, NO, and H_2_O_2_) and the decreased TAC and anti-oxidant serum levels (CAT, GPx, and GR) in CMG. Oxidative stress was a common finding in cow mastitis [15,22–24].

The oxidative stress observed in CMG clarified the increased renal and hepatic function tests in them. As the liberated free radicals during the disease course attack the kidney and liver cells, causing their damage. Consequently, urea and Cr accumulated in the diseased animals` blood due to renal damage, and liver enzymes (ALT, AST, GGT, and ALP) leaked into the mastitic animals` circulation due to hepatocyte destruction [15,25,26]. No doubt, renal insufficiency and the inability to restore minerals and electrolytes from urine played a role in the recorded decrease in mineral and electrolyte levels in CMG. Hepatic damage is also incorporated in the detected hypoalbuminemia, hypoglycemia, and T/LDL/HDL-hypocholesterolemia in CMG. As the liver is the organ concerned with albumin and cholesterol formation and serum glucose level regulation [27].

The invigorated pro-inflammatory cytokines greatly contribute to the systemic signs of the disease. They encourage vasodilators (histamine), prostaglandin E2, and bradykinin synthesis, resulting in udder congestion and swelling, pain sensation, pyrexia, and subsequent anorexia. Obviously, anorexia is responsible for hypoalbuminemia, hypoglycemia, T/LDL/HDL-hypocholesterolemia, decreased electrolytes, minerals, and trace elements blood concentrations in CMG. On the other hand, the reported hypertriglyceridemia referred to increased body fat lipolysis to get energy [15,23,25]. It is worth mentioning that the noted decline in the trace elements (Cu, Zn) in CMG in this data may be involved in the oxidative stress recorded in CMG. Cu and Zn are essential components in enzymatic antioxidant formation, and their deficiency means lower levels of antioxidants and oxidative stress amplification [15,23,25].

The protein profile of CMG in the present study was a mirror of the immune response. It showed prominent hypergloulinemia, dependent hyperproteinemia, and decreased A/G. The hyperglobulinemia pointed to different immune molecules production, such as cytokines, APPs, and matrix metalloproteinases which are α and β globulins (non-specific immunity), and immunoglobulins, which are γ globulins (specific immunity). These molecules are responsible for invading pathogens destruction and removal from the host body. Similar data were reported before by Sadat et al. [15] in bovine subclinical and clinical mastitis. On the contrary, notable hypoalbuminemia was determined in CMG. In addition to the above-mentioned anorexia, albumin is a negative APP that usually decreases during infections. It is mainly produced by hepatocytes, and in cases of infection, the liver ignores its production and prioritizes α and β globulins production [25].

Concerning the CMG hemogram in this work, it illustrated microcytic hypochromic anemia. This anemia was also attributed to pro-inflammatory cytokine activation. As pro-inflammatory cytokines stimulate iron trapping by macrophages, interfere with intestinal iron absorption, promote hepcidin production (compete with Tf for iron), and enhance ferritin formation. This action is mainly to prevent iron access to the pathogens and inhibit their multiplication and growth. Unfortunately, this action reduces iron availability in the host bone marrow and inhibits normal erythropoiesis in the animal, resulting in recorded anemia in CMG [12–16]. In addition, free radicals corrupt the RBC membranes’ integrity, leading to their lysis and resulting in decreased RBCs, Hb, PCV, and subsequent anemia in CMG [15,25,26]. In accordance, the iron profile of the CMG displayed obvious hypoferremia, hypotransferrinemia, and hyperferritinemia due to the above-mentioned iron sequestration mechanism. In addition, the acute phase response detected in CMG participated in this hypotransferrinemia, and hyperferritinemia, as Tf is a negative acute phase reactant and ferritin is a positive acute phase reactant [16,20–22]. Furthermore, oxidative lysis of RBCs causes free iron leakage and stimulates ferritin formation to avoid the free iron oxidative effect [15,22–24]. Logically, the increased TIBC and UIBC as well as the decreased Tf. Sat.% in CMG are consequences of the depicted hypoferremia.

The stated neutrophilia, eosinophilia, monocytosis, and subsequent leukocytosis in CMG in our data were also assigned to the activation of the pro-inflammatory cytokine. Pro-inflammatory cytokines induce different types of leukocyte creation, proliferation, maturation, and release from the bone marrow. The stress related to the disease also increases endogenous corticosteroids, which cause neutrophilia and leukocytosis [23,26]. The neutrophils are important in engulfing bacterial pathogens, and monocytes remove bacterial and tissue debris. On the other hand, the outstanding lymphocytopenia may be because of the increased blood cortisol levels, which act on the redistribution of circulating lymphocytes [26].

Regarding the estimated pro-inflammatory cytokines, APPs, and TAC value as prognostic and diagnostic tools for camel mastitis, their sensitivity, specificity, PPV, NPV, and AR suggested their use as excellent biomarkers for the disease, and LR-nominated TAC, Fb, and ferritin as the best markers among them, while the percentages of increase calculation cleared that IL-1α and IL-1β were the best. This suggests the combination of more than one marker for an accurate diagnosis. These results agreed with previous researchers, who considered oxidative stress biomarkers, APPs, and pro-inflammatory cytokines reliable indicators for mastitis in camels and other species. They mentioned their accuracy and correlation with other biochemical and immunological changes [11–15,19–24]. The observed differences in marker preference between the current study and previous research may be attributed to variations in factors such as disease stage, age, animal species, and other potentially confounding variables. It is important to consider these factors when comparing results across different studies in order to accurately interpret and generalize the findings. Furthermore, future research should aim to address these potential sources of variability in order to more accurately elucidate the underlying biological mechanisms and clinical implications of the observed marker preferences.

## Conclusion

Because of the opportunistic nature of the mastitic bacteria, preventive measures should be taken to reduce the risk of camel mastitis, such as ensuring proper hygiene during milking, identifying and treating any injuries or blockages in the milk ducts, providing good nutrition, and avoiding overmilking or undermilking the camel. When treating mastitis in she-camels, it is important to consider the associated alterations in immune response, clinicopathological parameters, and iron profile. TAC, Fb, ferritin, IL-1α, and IL-1β are valuable indicators for camel mastitis, but larger-scale studies on these markers are recommended.

## List of Abbreviations

A/G, Albumin/Globulin ratio; Alb, Albumin; ALP, alkaline phosphatase; ALT, alanine aminotransferase; AST, aspartate aminotransferase; AUC, area under the curve; B, Basophils; CAT, catalase; CG, control group; CMG, clinical mastitic group; Cp, caeruloplasmin; Cr, creatinine; E, Eosinophils; Fb, fibrinogen; GGT, gamma-glutamyl transferase; Glob, Total globulin; GPx, glutathione peroxidase; GR, glutathione reductase; H_2_O_2_, hydrogen peroxide; Hb: Hemoglobin concentration; HDL-c, HDL-cholesterol; Hp, haptoglobin; IL-1α, interleukin 1 alpha; IL-1β, interleukin 1 beta; IL-6, interleukin 6; IL-10, interleukin 10; L, Lymphocytes; LDL-c, LDL-cholesterol; LR, likelihood ratio; M, Monocytes; MCH, Mean corpuscular hemoglobin; MCHC, Mean corpuscular hemoglobin concentration; MCV: Mean corpuscular volume; MDA, malondialdehyde; N, Neutrophils; NO, nitric oxide; NPV, negative predictive value; PCV: Packed cell volume; PPV, positive predictive value; RBCs, Red blood cell count; SAA, serum amyloid A; SI, Serum iron; TAC, total antioxidant capacity; Tf, Transferrin; Tf sat.%, Transferrin saturation percentage; TIBC, Total iron binding capacity; TLC, Total leukocytic count; TNF-α, tumor necrosis alpha; TP, Total protein; UIBC, Unsaturated iron binding capacity.
